# Overcoming physiological trade-offs between flowering time and crop yield: strategies for a changing climate

**DOI:** 10.1093/jxb/eraf110

**Published:** 2025-03-11

**Authors:** Astrid Wingler, Soualihou Soualiou

**Affiliations:** School of Biological, Earth & Environmental Sciences and Environmental Research Institute, University College Cork, Distillery Fields, North Mall, Cork T23 TK30, Ireland; School of Biological, Earth & Environmental Sciences and Environmental Research Institute, University College Cork, Distillery Fields, North Mall, Cork T23 TK30, Ireland; University College Dublin, UK

**Keywords:** Crop yield, flowering time, harvest index, inflorescence photosynthesis, nitrogen-use efficiency, phenology, resource allocation, senescence, source–sink, stay-green

## Abstract

Early flowering of annual plants can lead to resource limitation owing to reduced uptake of nitrogen during the reproductive phase and declining foliar photosynthesis as a result of monocarpic senescence. Low availability of accumulated resources can therefore result in a trade-off between early flowering and reproductive fitness. However, green inflorescence organs (such as siliques, pods, bracts, or awns) can make considerable contributions to photosynthetic carbon gain, and in some cases provide more carbon to seed formation than the leaves. Inflorescence photosynthesis may thereby overcome the flowering time trade-off. In addition to providing photosynthates, inflorescence organs can contribute to seed nitrogen through senescence-dependent nitrogen recycling. In annual crops, breeding has resulted in increased carbon allocation to the grain and higher harvest index, but in some cases this had led to reduced grain protein content. We discuss different breeding targets to address carbon and nitrogen limitation, dependent on the climatic environment. For environments that are prone to drought, we propose a combination of early flowering with enhanced inflorescence photosynthesis or, alternatively, delayed senescence (stay-green) associated with improved water balance. For optimized yield and grain protein content under favourable conditions, enhanced sink strength and extended nitrogen uptake are suggested as breeding targets.

## Teaser

This review describes strategies for how to improve annual crop yield by optimizing flowering time, inflorescence photosynthesis, and senescence, dependent on the climatic conditions.

## Introduction

Plant growth and reproduction can be limited by the acquisition of resources, such as photosynthetic carbon assimilation or nitrogen uptake (source limitation), or by the utilization of resources for the growth and development of sinks (sink limitation) (White *et al.*, 2016). The allocation of photosynthates and nutrients from source to sink plays an important role in the growth and reproduction of wild plants under natural conditions, while in agricultural systems resource allocation (e.g. during grain filling) determines crop yield and quality (Griffiths *et al.*, 2016; Ludewig and Sonnewald, 2016; Paul *et al.*, 2020).

Flowering time has a significant impact on resource acquisition and allocation of plants, in addition to being affected by climate change. For example, the timing of floral transition in response to drought stress has recently been identified as one of the ‘burning questions for a warming and changing world’ (Verslues *et al.*, 2023). In wild plants, early flowering in response to stress such as drought (drought escape) can be an adaptive response, enabling the plants to recycle their nutrients and complete their life cycle before terminal drought later in the season (Monroe *et al.*, 2018; Hwang *et al.*, 2019). In crops such as cereals, early flowering can also be beneficial under hot and dry conditions (Barnabás *et al.*, 2008; Shavrukov *et al.*, 2017). However, early flowering may not allow plants to accumulate sufficient biomass and resources (carbon and nitrogen) which could later be remobilized for grain production. Therefore, late flowering may be beneficial under more favourable conditions without terminal drought (Gol *et al.*, 2017).

**Fig. 1.**
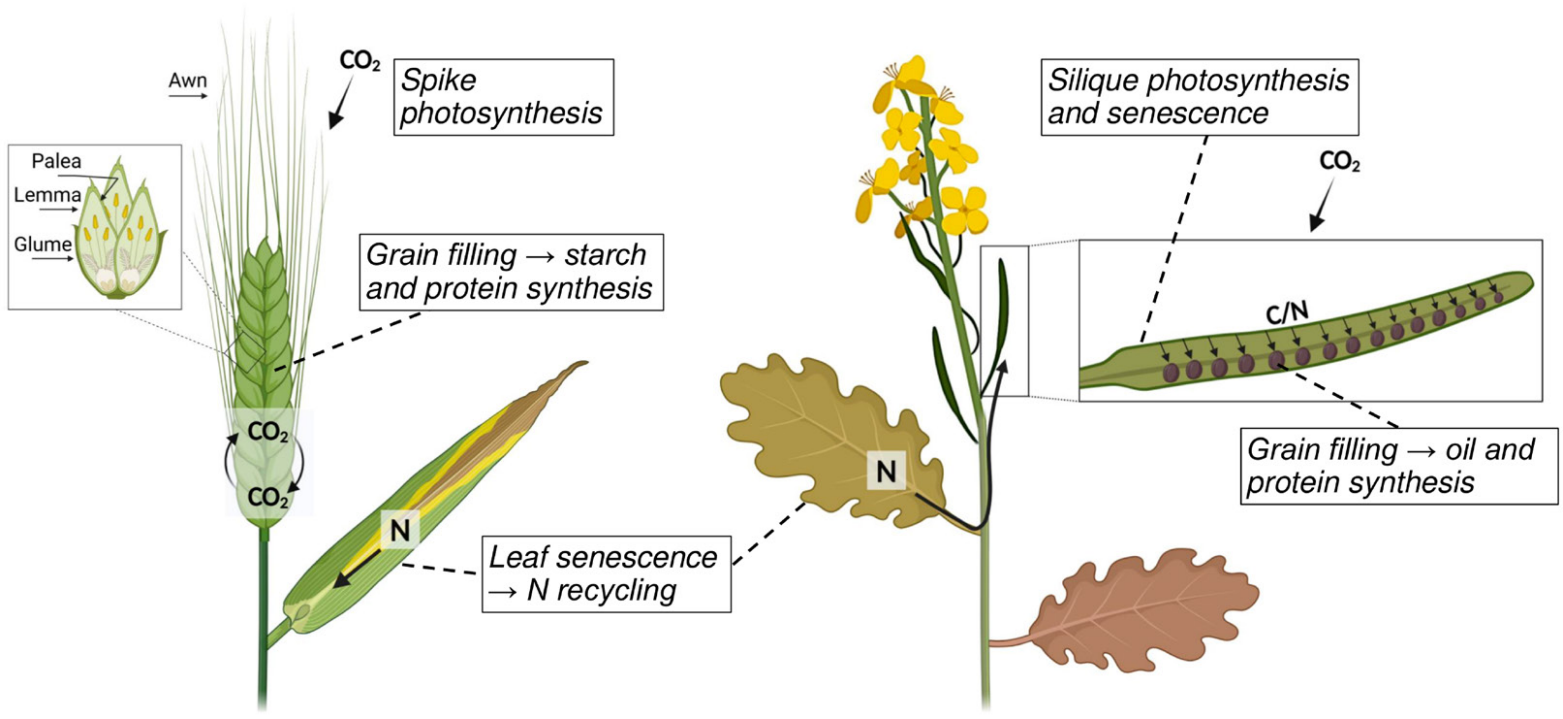
The role of inflorescence photosynthesis and senescence-dependent nutrient recycling. In cereals (left), awns and bracts (glume, palea, and lemma) play an important role in the provision of photosynthates to the grain, by uptake of external CO_2_ and refixation of respired CO_2_ by the bracts. In oilseeds (right), silique wall photosynthesis provides carbon for grain filling. Senescence-dependent recycling of nitrogen from the leaves is important for the formation of the photosynthetic apparatus in the inflorescence and can later be recycled from the inflorescence for grain protein synthesis. Created in BioRender. Wingler, A. (2025) https://BioRender.com/h83u256.

By contributing to photosynthetic carbon gain for grain filling later in development, enhanced inflorescence photosynthesis may be able to overcome the trade-off between early flowering and grain yield. This was demonstrated using the model plant Arabidopsis (*Arabidopsis thaliana*) where inflorescence photosynthesis can make a considerable contribution to photosynthetic carbon gain, allowing early flowering plants to maintain high reproductive fitness despite small vegetative size (Gnan *et al.*, 2017). It has been demonstrated that inflorescence photosynthesis also makes an important contribution to carbon gain in crops such as oilseeds and cereals (e.g. Raven and Griffiths, 2015; Sanchez-Bragado *et al.*, 2020; Wang *et al.*, 2023; Vincente *et al.*, 2024) (Fig. 1). Potential source limitation can therefore be overcome not just by higher leaf photosynthesis, but also by photosynthesis of green inflorescences, including floral organs (such as siliques, pods, bracts or awns), inflorescence stems, and green seeds (Brazel and Ó’Maoiléidigh, 2019).

Flowering time also determines the allocation of photosynthates to the grain, and is therefore associated with harvest index (HI; i.e. the ratio of grain biomass to total shoot biomass) (Chardon *et al.*, 2014). During the 20th century, breeding has increased HI (Sinclair, 1998), resulting not only in increased yields but also in improved response to nitrogen fertilizer application in green revolution varieties. However, a theoretical HI limit of 0.6 has been reached for some crops, such as wheat (*Triticum aestivum*) and rice (*Oryza sativa*) (Rose and Kage, 2019; Furbank *et al.*, 2020). In addition, high nitrogen availability is essential to support yield in crops with high HI (Sinclair, 1998), and high HI can be associated with lower grain protein content (GPC) and thus lower quality (Prey *et al.*, 2019a; Maeoka *et al.* 2020; Hernández-Ochoa *et al.*, 2023). GPC is also dependent on senescence-related nitrogen recycling from the leaves (Distelfeld *et al.*, 2014) and post-anthesis root nitrogen uptake (Prey *et al.*, 2019b; Nehe *et al.*, 2020). The factors that determine carbon and nitrogen acquisition and allocation, such as flowering time, leaf or inflorescence photosynthesis, nitrogen uptake, and senescence, therefore have to be considered together in attempts to optimize crop yield and quality dependent on the climatic conditions.

## Flowering as determinant of harvest index and yield

Flowering time, a major phenological event, directly influences the duration of vegetative growth and the allocation of resources towards reproductive structures (Obeso, 2002; Metcalf *et al.*, 2003; Haselhorst *et al.*, 2011). An optimal flowering time ensures a balanced source–sink relationship, leading to efficient translocation of photosynthates to the grains. If flowering occurs too early, monocarpic senescence limits carbon gain, and root nitrogen uptake capacity declines (Rossato *et al.*, 2001). A short vegetative phase can therefore limit grain yield due to insufficient resource accumulation (Gol *et al.*, 2017). Conversely, late flowering might result in a shortened grain-filling period, reducing grain size and overall yield. However, provided the grain-filling period remains adequately long, an extended vegetative phase before flowering can enhance biomass production and subsequently increase grain yield (Fischer and Stockman, 1986).

Experiments with the model species Arabidopsis suggest that there is a genetic basis that determines the relationship between flowering time, reproduction and HI: a quantitative trait locus (QTL) meta-analysis of three Arabidopsis recombinant inbred line populations revealed an overall negative correlation of flowering time with HI (in two out of three recombinant inbred line populations), and a stronger negative correlation of flowering time with the ratio of reproductive to vegetative organ weight (in all three populations) (Chardon *et al.*, 2014). This suggests that early flowering plants allocate more resources to reproduction. Previously, early flowering and senescence were linked to higher fecundity for Arabidopsis accessions (Levey and Wingler, 2005), confirming that there is a compensatory mechanism that overcomes the shortage of accumulated resources in early flowering genotypes. This mechanism is likely to be genetically determined as smaller Arabidopsis genotypes have higher fecundities (Aarssen and Clauss, 1992; Ferguson *et al.*, 2018). However, the impact on environmental variation is opposite, with larger individuals of the same genotype having higher fecundities (Aarssen and Clauss, 1992).

Inflorescence photosynthesis, which can contribute >90% of plant photosynthetic activity in Arabidopsis (Leonardos *et al.*, 2014), can support the reproductive performance of early flowering genotypes and overcome the limitation of small reproductive size at flowering. Gnan *et al.* (2017) showed that, even if all rosette leaves are removed at flowering, Arabidopsis accessions are still able to maintain reproductive fitness: on average, seed formation decreased by 40%, but some accessions showed little impact of rosette leaf removal.

For crops, it has also been shown that the trade-off between early flowering and grain yield can be broken. Maeoka *et al.* (2020) found that semi-dwarf winter wheat varieties yielded more due to a shorter vegetative period and longer grain filling, which increased the number of grains per ear and dry matter allocation to grains, thus improving the HI. These varieties also responded positively to fertilization, showing higher nutrient concentrations (nitrogen, phosphorus, potassium, and sulfur) in the grains. Importantly, the early flowering trait can modify the dynamics of nutrient uptake and allocation, allowing more biomass and nutrients to be allocated to grains. On the other hand, there are limitations to this approach in crops. Xi *et al.* (2023) described for spring wheat genotypes that the relationship between flowering time and yield is unimodal. While higher yields and a higher reproductive effort have resulted from earlier flowering, optimum flowering time has already been achieved and additional advancements in flowering could result in decreased yield. It was also shown that early flowering caused a reduction in root length density and total leaf area, thus inhibiting the capacity of vegetative structures to acquire enough resources, which are required later to support higher grain yield (Fletcher *et al.*, 2015).

In addition, environmental conditions affect the intricate balance between flowering time and yield. Optimizing flowering time can maximize grain yield by ensuring that the plant experiences optimal environmental conditions, such as temperature and moisture, during critical growth periods (Sadras and Dreccer, 2015; Slafer *et al.*, 2015). For instance, flowering too early in the season can expose the plant to late frosts or insufficient warmth, potentially reducing biomass accumulation and grain filling. Conversely, flowering too late can lead to heat or drought stress during the grain-filling stage, which can also diminish yield (Craufurd and Wheeler, 2009; Johansson *et al.*, 2013). For example, Zhang *et al.* (2021) illustrated for wheat cultivars in diverse environments that synchronizing flowering with periods of moderate temperatures and adequate water availability enhanced grain yield.

## Contribution of inflorescence photosynthesis to grain filling in crops

In line with the contribution of inflorescence photosynthesis to seed production in Arabidopsis (Leonardos *et al.*, 2014; Gnan *et al.*, 2017; Zhu *et al.*, 2018), it also plays an important role in grain filling in the related crop, oilseed rape (*Brassica napus*) (Fig. 1; Table 1). In particular, the silique wall contributes to photosynthetic carbon gain and thereby seed oil content. Its photosynthetic contribution increases with development and can exceed leaf photosynthesis because of higher irradiance intercepted at the top of the canopy (Gammelvind *et al.*, 1996). Shading of siliques therefore reduced seed yield and oil content substantially in field-grown oilseed rape plants (C. Wang *et al.*, 2016). Recently, the remarkable influence of silique photosynthesis has been confirmed: shading from the end of flowering to seed ripening revealed an average contribution of 84% and 21% to seed yield and oil content, respectively, compared with the non-shading control treatment (Table 1) (Wang *et al.*, 2023). Similar to the role of silique wall photosynthesis, a contribution of pod photosynthesis to seed weight and composition was shown for soybean (*Glycine max*), suggesting that non-foliar photosynthesis is also important in legume crops (Cho *et al.*, 2023). By covering pods, the authors demonstrated that 13–14% of mature seed weight was produced by pods, with pod and seed photosynthesis contributing 9% of total daily carbon gain. Previous research had shown that pod shading only affects soybean seed weight in combination with defoliation (Bianculli *et al.*, 2016), suggesting that pod photosynthesis mainly plays a role during source limitation.

**Table 1.** Contribution of inflorescence photosynthesis to yield parameters in different plant species across various growing conditions

Species	Treatment	Inflorescence/inflorescence organ involved	Estimated contribution to yield	References
*Arabidopsis thaliana*	Ambient and elevated CO_2_	Whole inflorescence	90% (to daily carbon gain/biomass)	Leonardos *et al.* (2014)
*Arabidopsis thaliana*	Normal	Whole inflorescence	55% (lifetime carbon gain)	Earley *et al*. (2009)
Oilseed rape (*Brassica napus*)	Shading	Silique	54% (to grain yield per plant) and 42% (to seed oil content)*	C. Wang *et al.* (2016)
Oilseed rape (*Brassica napus*)	Shading	Silique	84% (to grain yield per plant) and 21% (to seed oil content)*	Wang *et al.* (2023)
Oilseed rape (*Brassica napus*)	Girdling	Silique wall	43% (to seed oil content)	Hua *et al.* (2012)
Soybean (*Glycine max*)	Pod shading. Pod shading and defoliation	Pod	No effect. 75% (to individual seed weight) and 15% (to seed oil concentration)*	Bianculli *et al.* (2016)
Soybean (*Glycine max*)	Shading	Pod	13–14% (to individual seed weight) and 2–5% (to seed oil concentration)	Cho *et al.* (2023)
Alfalfa (*Medicago sativa/M. varia*)	Shading	Pod	26–48% (to seed weight per pod)	H. Wang *et al.* (2016)
Rice hybrid (*Oryza sativ*a)	Gibberellin application	Panicle	42% (to grain yield)	Zheng *et al.* (2018)
Rice backcross line (*Oryza sativ*a *japonica*/*O. rufipogon*)	Normal	Awn	4% (to grain yield per plant)*	Jin *et al.* (2009)
Wheat (*Triticum aestivum*)	Shading	Ear	60% (to grain weight per ear)	Sanchez-Bragado *et al.* (2016)
Wheat (*Triticum aestivum*)	Defoliation Rainfed and shading (leaves and stems)	Ear	44% (to grain weight per ear) 29% (to grain weight per ear)	Maydup *et al.* (2010)
Wheat (*Triticum aestivum*)	Rainfed versus irrigation (R and I); fertilized versus non-fertilized (F and NF)	Ear	55% under R+F (to grain nitrogen gain) 34% under R+NF (to grain nitrogen gain) 42% under I+F (to grain nitrogen gain) 38% under I+NF (to grain nitrogen gain)	Sanchez-Bragado *et al.* (2017)
Wheat (*Triticum aestivum*)	Terminal heat stress	Ear Awn	2–46% for ear (to grain weight of main ear) 5–30% for awns (to grain weight of main ear)	Pradeep *et al.* (2024)
Wheat (*Triticum aestivum*)	High nitrogen versus low nitrogen; spikelet removal	Spikelet	61% under high nitrogen (to grain dry weight per culm) 58% under low nitrogen (to grain dry weight per culm)	Wu *et al*. (2022)
Wheat NILs (*Triticum aestivum*)	Awned versus awnless	Awn	24% for awns (to grain yield per plant)*	Wang *et al.* (2020)
Wheat genotype panels (*Triticum aestivum*)	Yield potential/optimum Heat stress	Ear Ear	33% (to grain weight per ear) 34% (to grain weight per ear)	Molero and Reynolds (2020)
Wheat (*Triticum aestivum*)	Rainfed Irrigated	Awned Awnletted Awned Awnletted	67% (to ear harvest index) 72% (to ear harvest index) 69% (to ear harvest index) 74% (to ear harvest index)	Rebetzke *et al.* (2016)
Wheat (*Triticum aestivum*)	De-awning; rainfed	Awn	10% (to grain yield per plant)*	Khaliq *et al.* (2008)
Wheat (*Triticum aestivum*)	Irrigated Dry	Awned Awnless Awned Awnless	34% (to grain carbon) 13% (to grain carbon) 43% (to grain carbon) 24% (to grain carbon)	Evans *et al.* (1972)
Durum wheat (*Triticum turgidum* var. *durum*)	Shading (ears and stem) Defoliation/de-awning	Ear Awn	24–26% for ear (to 1000 kernel weight) 4–15% for awns (to 1000 kernel weight)	Merah *et al.* (2018)
Durum wheat (*Triticum turgidum* var. *durum*)	Shading (flag leaf or ear)	Ear	41% (to grain weight per ear)	Araus *et al.* (1993)
Durum wheat (*Triticum turgidum* var. *durum*)	Defoliation Defoliation and shading (stem and leaves)	Awn Ear	9% for awns (to 1000 kernel weight) 22.5% for ear (to 1000 kernel weight)	Merah and Monneveux (2015)
Durum wheat (*Triticum turgidum* var. *durum*)	Ear shading and awn excision; irrigation	Ear Awn	35–60% for ear (to grain weight per ear) 11–14% for awns (to grain weight per ear)	Arous *et al.* (2020)
Barley (*Hordeum vulgare*)	Irrigated	Awn	30% (to grain yield per plant)*	Hosseini *et al.* (2012)
Barley (*Hordeum vulgare*)	Awned versus awnless; rainfed	Awn	13% for awns (to grain yield per plant)*	Schaller and Qualset (1975)
Barley NILs (*Hordeum vulgare*)	Awned versus awnless; shading of ears	Awned Awnless	38% (to grain weight per ear) 15% (to grain weight per ear)	Bort et *al*. (1994)
Siberian wildrye (*Elymus sibiricus*)	De-awning; irrigated and rainfed	Awn	No significant effect on grain yield per plant or 1000 seed weight	Ntakirutimana et *al*. (2021)

If the referenced publications reported yield contributions for more than one crop variety or season, means were calculated from these data. In cases where no contributions to yield were given in the references, the values were calculated (marked with *) from the yield parameters presented in the tables and figures.

The photosynthetic contribution of inflorescences has been analysed in most detail for cereal crops, where it was shown to be a crucial factor in yield production, for example in wheat, barley (*Hordeum vulgare*), and rice (Sanchez-Bragado *et al.*, 2020). Traditionally, the leaves, especially the flag leaves, have been considered the primary source of photosynthates during the grain-filling period. However, several studies have highlighted a significant role for inflorescence photosynthesis in contributing to cereal yield. The inflorescence (the ear in, for example, wheat and barley; or the panicle in, for example, rice) has green organs, including bracts (glumes, lemma, and palea) and—dependent on genotype—awns. These organs can perform photosynthesis and contribute to varying degrees to the overall carbon gain (Brazel and Ó’Maoiléidigh, 2019; Tambussi *et al.*, 2021; Lawson and Milliken, 2023; Sanchez-Bragado *et al.*, 2023) (Table 1). Photosynthetic activity of the inflorescence becomes particularly important during the later stages of grain development when the contribution from the leaves may decline due to shading, senescence, or environmental stresses such as drought or high temperatures (Tambussi *et al.*, 2007; Sanchez-Bragado *et al.*, 2020). Accordingly, Araus *et al.* (2021), using large-scale data collected on various crop species (including rice, barley, and wheat), reported inflorescence CO_2_ assimilation values between 0.6 μmol m^–2^ s^–1^ and 30.3 μmol m^–2^ s^–1^ dependent on the tissue and experimental conditions, reflecting between 10% and 600% of flag leaf rates. Additionally, the contribution of ear and panicle photosynthesis to yield was estimated to range from 10% to 59% of photosynthates deposited in grains of major cereals, dependent on cultivar and experimental conditions (Hu *et al.*, 2019).

Ear photosynthesis has several distinct advantages that enhance its contribution to yield. Firstly, the proximity of photosynthetically active tissues to the developing grains means that the photosynthates can more efficiently be transported to the grains. ^14^C-labelling showed that, compared with photosynthates from the flag leaf, photosynthates from the ear are exported and distributed more uniformly within the spikelet and between grains (Rawson and Evans, 1970). Additionally, the ear can continue to photosynthesize under conditions where leaf photosynthesis is compromised. For example, in wheat subjected to drought stress, the ears often have a more persistent photosynthetic apparatus than the flag leaf, providing a sustained source of assimilates as the amounts of chlorophyll, Rubisco, and light-harvesting complex II (LHCII) protein are maintained in bracts and awns (Martinez *et al.*, 2003). A study by Maydup *et al.* (2010) revealed that ear photosynthesis makes a significant contribution to grain yield of wheat, from 13% to 33% in the absence of stress, and up to 22–45% under defoliation or water deficit. A higher net photosynthesis rate (estimated on an area basis) was induced in the ear compared with the flag leaf under unfavourable growth conditions. This provides evidence that ear photosynthesis has a capacity to safeguard grain filling when leaf photosynthesis is constrained by stress.

Moreover, the position at the top of the canopy allows ears to intercept sunlight. For example, the ears of awned wheat cultivars intercepted a similar proportion of light (30%) to the flag leaves (Sanchez-Bragado *et al.*, 2014). The presence of the awns can therefore expand the photosynthetic area of the ear and the overall photosynthetic activity of the plant (Sanchez-Bragado *et al.*, 2023). The capacity of awns to intercept light and their long green duration until grain maturity support a key role in carbon gain during the final stages of development. The role of awn length and presence has been investigated in detail in wheat. Wang *et al.* (2020) used the natural variation in wheat accessions to identify a promoter of *Awn Length Inhibitor 1* (*ALI-1*), which is associated not only with awn length, but also grain length and weight. These findings indicate a contribution of awns to source and sink strength simultaneously, which could improve yield.

Although Sanchez-Bragado *et al.* (2023) acknowledge the positive impact of awns on grain weight, they note a limitation of awn photosynthesis in improving yields. Yield data from previous research showed no clear support that awned near-isogenic lines outperform their awnless counterparts, and yield benefits/penalties were independent of the presence of awns. While the presence of awns increases grain weight, it decreases grain number (Rebetzke *et al.*, 2016; Sanchez-Bragado *et al.*, 2023). This contrasting effect of awns may be explained with the finding that awns are sinks for photosynthates at the same time as when these photosynthates are also critical for floret development (Zhang *et al.*, 2021). If the rate of development and awn size increase with favourable growing conditions, this can result in higher resource limitation for the floret development and therefore reduce ear fertility and the number of grains (Rebetzke *et al.*, 2016).

Among the various characteristics that make ear photosynthesis valuable for increasing yields, the refixation of CO_2_ respired by developing grains is another important factor. Ear bracts bear a greater capacity (compared with leaves and awns) to refix CO_2_ released from developing grains due to their anatomical structure and positioning (Bort *et al.*, 1996; Tambussi *et al.*, 2007). The physiological implications of this refixation process include not just the prevention of respiration losses but also promotion of water-use efficiency in the ears, regardless of external gas exchange. In C_3_ cereals, such as wheat, barley, and rice, respiratory CO_2_ significantly influences photosynthesis, with 55–75% of respired CO_2_ being refixed, which substantially contributes to grain yield (Bort *et al.*, 1996; Sanchez-Bragado *et al.*, 2020; Zhang *et al.*, 2022). Refixation of respiratory CO_2_ plays an important role as an extra source of carbon in the ear, as atmospheric carbon fixation can be limited either by constraints in stomatal diffusion or by reduced light penetration (Simkin *et al.*, 2020).

Overall, the photosynthetic contribution of inflorescences in annual plants is critical to yield production as it complements the role of leaf photosynthesis and ensures a steady supply of assimilates under various growing conditions.

## Importance of nitrogen-use efficiency for harvest index and grain protein content

In the context of high fertilizer costs and the need to reduce nitrogen pollution, including production of the greenhouse gas N_2_O, improving nitrogen-use efficiency (NUE) is a high priority. NUE can be expressed as grain yield per unit of nitrogen fertilizer applied or as the fraction of fertilizer nitrogen that is allocated to yield (Congreves *et al.*, 2021). It can be improved by addressing nitrogen uptake efficiency and/or nitrogen utilization efficiency, the latter reflecting nitrogen assimilation efficiency and nitrogen remobilization efficiency (Masclaux-Daubresse *et al.*, 2010).

When nitrogen supply is limited, there can be a trade-off between the allocation of nitrogen to the photosynthetic apparatus in the leaves for carbon assimilation and investment of nitrogen in seeds for reproductive fitness (wild plants) or grain filling (crops). Sinclair (1998) revised the concept of HI by inclusion of nitrogen as a crucial resource that must be remobilized to support the relative change in grain mass. Since in some crops, such as wheat, the nitrogen concentration in the grain is much higher than that in the straw, an increase in the relative fraction of grain necessitates an increase in nitrogen accumulation by the plant. Sinclair (1998) therefore proposed that a low HI may be advantageous under low nitrogen availability. The view that high HI results in nitrogen limitation is also supported by the finding that high yield and high HI in modern cultivars and hybrid wheat are associated with lower GPC (Prey *et al.*, 2019a; Maeoka *et al.*, 2020; Hernández-Ochoa *et al.*, 2023). This supports previous findings for Arabidopsis recombinant inbred line populations, showing that HI is negatively correlated with seed nitrogen content (Chardon *et al.*, 2014).

**Fig. 2.**
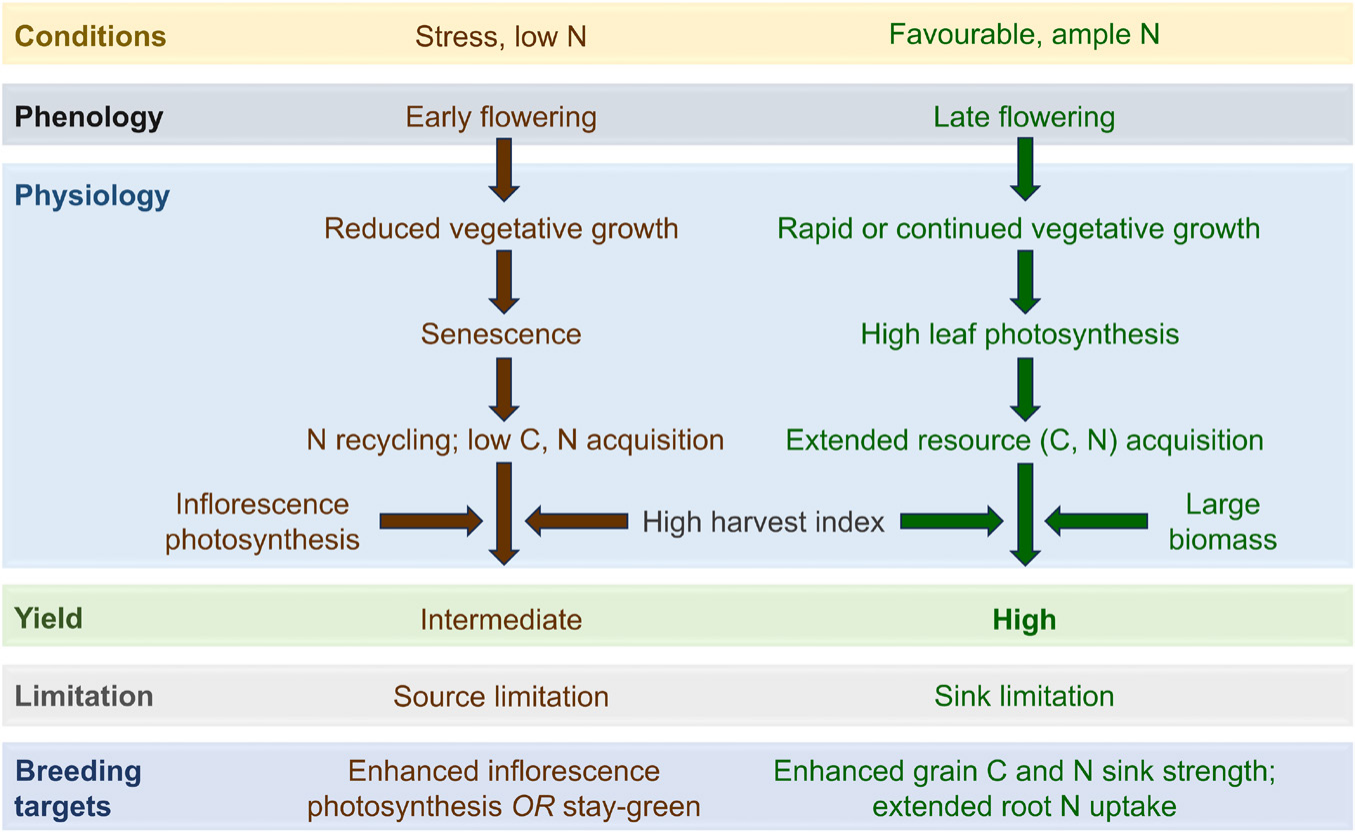
Proposed breeding targets for improved grain yield and quality under stress and favourable conditions. The high harvest index of modern cultivars already supports yields but can result in nitrogen limitation. Under stress conditions (left, brown), early flowering can be beneficial (stress escape). Senescence is important for nitrogen recycling, especially under low-nitrogen conditions. Enhanced inflorescence photosynthesis can compensate for the shorter vegetative photosynthetic period resulting from earlier leaf senescence. Alternatively, stay-green of leaves can improve yield under source limitation if it is associated with improved water balance. Under favourable conditions (right, green), high leaf photosynthetic activity and late senescence support high source strength but can result in sink limitation. Enhanced grain sink strength and extended post-anthesis nitrogen uptake are therefore proposed as the main breeding targets not just to support grain yield but also to overcome the trade-off between high harvest index and grain protein content.

To reach the target of combining high HI with high GPC, nitrogen allocation within the plant (nitrogen utilization efficiency) needs to be improved in combination with nitrogen accumulation by the plant (nitrogen uptake efficiency), especially if fertilizer use is supposed to be decreased. Both nitrogen uptake and utilization efficiency have been shown to be important for wheat yield (Prey *et al.*, 2019a, b). Post-anthesis uptake of nitrogen from the soil is associated with high flag leaf photosynthesis, high biomass, high yield, and high NUE in wheat (Prey *et al.*, 2019b; Nehe *et al.*, 2020). It not only increases nitrogen availability for grain filling, but also allows extended photosynthetic carbon acquisition and has the potential to meet the increased nitrogen requirement in crops with high HI (Fig. 2). Leaf senescence, on the other hand, limits photosynthetic life span but is important for the remobilization of nitrogen to the grain and thereby nitrogen utilization efficiency. However, a trade-off has been described between nitrogen remobilization from leaves and post-anthesis nitrogen uptake, which could limit GPC (Havé *et al*, 2017).

## Leaf senescence and stay-green as alternative approaches to support nitrogen-use efficiency and yield

While high NUE has generally been optimized for cereals such as wheat, it is lower in oilseed rape, resulting in considerable loss of nitrogen to the environment. Although nitrogen from vegetative organs already makes an important contribution to grain filling (Malagoli *et al.*, 2005a), modelling shows that nitrogen HI can be improved by increasing nitrogen transfer from vegetative to reproductive tissues (Malagoli *et al.*, 2005b). In addition, nitrogen recycling from the lower leaves to the leaves higher up in the oilseed rape canopy is restricted by early abscission, and longer duration of these lower leaves may be required to optimize senescence-dependent nitrogen recycling (Malagoli *et al.*, 2005a). These findings indicate that nitrogen recycling from vegetative tissues, including leaves and stems, is a limiting factor for crops such as oilseed rape (Malagoli *et al.*, 2005a, b), and similarly for maize (Mueller *et al.*, 2019). However, delayed senescence combined with post-anthesis uptake of nitrogen from the soil has also been shown to play a major role in NUE of oilseed rape: while extended uptake of nitrogen can delay senescence, extended photosynthetic activity (stay-green), in turn, provides the photosynthates required for continued nitrogen uptake and silique development (He *et al.*, 2021).

The regulation of leaf senescence is also important for the recycling of nitrogen and other nutrients in cereal crops. Research on genetic variation in *Gpc-1*/*NAM-1* transcription factor genes has shown that early senescence results in more efficient nutrient remobilization and increased GPC in wheat and barley (Uauy *et al.*, 2006; Parrott *et al.*, 2012). However, yield and GPC are often negatively correlated (Distelfeld *et al.*, 2014). For example, crossing the wild emmer (*Triticum turgidum* ssp. *dicoccoides*) *Gpc-B1* allele for earlier senescence and higher GPC into wheat cultivars resulted in slightly lower yield. While early senescence limits photosynthetic leaf longevity, delayed senescence (functional stay-green) can extend the photosynthetic period and increase yield, as demonstrated, for example, for maize and sorghum, mostly under stress conditions (Gregersen *et al.*, 2013). However, yield is not always increased in stay-green genotypes, and in some cases decreased yield was observed. For example, despite leading to delayed senescence and higher rates of post-anthesis photosynthesis, RNAi repression of *NAM-B1* in wheat did not result in higher grain yield (Borrill *et al.*, 2015). Instead, limited starch synthesis during grain filling resulted in sink limitation. Similarly, green canopy duration did not positively affect grain filling in German winter wheat cultivars (Lichthardt *et al.*, 2020), confirming that the impact of stay-green traits on yield depends on source–sink interactions, with little effect if sink strength is not enhanced in tandem with the higher source strength resulting from the stay-green trait. In contrast, Luche *et al.* (2017) showed for Brazilian wheat that stay-green can increase yield not only under stress conditions, but also under favourable conditions through increasing ear fertility and the number of grains, thus enhancing source and sink strength.

Further, the impact of stay-green is more complex than simply affecting carbon and nitrogen dynamics. It can also result in increased root length, altered root architecture, and lower transpiration, thus improving drought tolerance, as shown for sorghum (Borrell *et al.*, 2014, 2022; Djanaguiraman *et al.*, 2024). Although modelling for winter wheat has suggested that stay-green could increase water use (Kothari *et al.*, 2019), the opposite was found for sorghum where the stay-green trait was associated with reduced canopy size at flowering and thus lower pre-anthesis water demand caused by leaf transpiration (Borrell *et al.*, 2014, 2022). Unexpectedly, stay-green in sorghum did not only affect pre-anthesis canopy size by reducing tillering and the size of the upper leaves (Borrell *et al.*, 2014), but leaf area was also reduced by earlier senescence of the lower leaves (George-Jaeggli *et al.*, 2017). By reducing canopy transpiration earlier on in the season, stay-green results in more water being available post-anthesis, provided the soil has a good water-holding capacity.

## Importance of nitrogen recycling from the inflorescence for grain filling and grain protein content

Most research on senescence has focused on leaf senescence, whereas the importance of inflorescence senescence for crop yield has received less attention. We propose here that efficient senescence-dependent recycling of nutrients, in particular nitrogen, from the inflorescences is an important target for improving grain yield and protein content (Fig. 1). For oilseeds, the timing of silique senescence was suggested as an important component in grain filling to increase the content of carbohydrates, lipids, and protein (Bennett *et al.*, 2011). For wheat, Sanchez-Bragado *et al.* (2020) highlight the contribution of ears to grain nitrogen. For example, Lopes *et al.* (2006) showed that losses of nitrogen in the flag leaf and glumes accounted for ~19% and 38%, respectively, of nitrogen in the grains, while Sanchez-Bragado *et al.* (2017) estimated that vegetative parts contribute 30–40% and the ear ~42% of grain nitrogen. For high-yielding wheat genotypes, Zhou *et al.* (2016) demonstrated that ears provided more Rubisco-derived nitrogen to the grain than flag leaves, but the opposite was the case in a low-yielding genotype. These findings suggest that the photosynthetic apparatus of ear organs is not just an important source of carbon for yield, but also plays a major role in contributing nitrogen to GPC.

Nitrogen allocation from the inflorescence organs requires senescence processes that recycle nitrogen from the photosynthetic apparatus, similar to those in leaves, including proteolysis, amino acid metabolism, and phloem export (Masclaux-Daubresse *et al.*, 2010). It is important that these processes operate efficiently and rapidly, but not too early as the late senescence of ears and other inflorescences is one of the key factors in extended photosynthesis, which allows carbon capture for grain filling (Sanchez-Bragado *et al.*, 2020; Tambussi *et al.*, 2021) and thereby contributes to stay-green at the canopy level.

## Old and new targets in breeding for increased grain yield

Yield can be increased by enhancing the overall acquisition of resources (increased rates of photosynthesis, increased photosynthetic life span, increased uptake of soil nutrients, and greater plant size), by altering the relative allocation of resources (remobilization of photosynthates and nutrients, higher HI), or a combination of these approaches. Which breeding approach may be successful depends on whether a crop is source or sink limited and on the environment (Fig. 2). Maydup *et al.* (2010) conclude that modern cultivars of wheat are becoming more source limited. This is supported by research showing that increased leaf photosynthesis is associated with larger plant biomass and higher wheat yield (Gaju *et al.*, 2016; Nehe *et al.*, 2020; Li *et al.*, 2023). Foulkes *et al.* (2011), in contrast, argue that wheat is mainly sink limited. To overcome sink limitation, it is proposed that breeding approaches should focus on traits that improve sink activity, such as increased ear fertility, grain number, and grain filling/size, in addition to HI (Foulkes *et al.*, 2011). Although breeding for higher HI has resulted in increased yields in the more recent past, higher grain yield during crop domestication was associated with greater plant size (Preece *et al.*, 2017). In current times, the relationship of HI with wheat yield may be decreasing instead of increasing, and breeding for increased total biomass may again become more important (Prey *et al.* 2019b; Rose and Kage, 2019). Nevertheless, HI was identified as an important determinant of grain yield in barley in Australia, with shoot biomass making a smaller contribution (Cossani *et al.*, 2022). Overall, high HI continues to be an important trait under a range of environmental conditions (Fig. 2).

The genetic control of flowering time presents significant opportunities for breeding programmes to optimize yield in crops, such as wheat (Reynolds *et al.*, 2009). Especially for Mediterranean climates, early flowering and drought escape can be successful strategies (Shavrukov *et al.*, 2017). Even under more benign conditions in the UK, it was shown that early flowering in combination with later onset, but faster rate of leaf senescence can enhance wheat grain filling, resulting in larger grains (Xie *et al.*, 2016). This enables extended photosynthetic and reproductive periods, but also rapid nutrient recycling during grain filling. If a combination of early flowering with a delayed onset of leaf senescence is desirable, it is necessary to uncouple these developmental processes. Barley high-GPC lines with early senescence are also early flowering. However, this is not necessarily the case for wheat (Distelfeld *et al.*, 2014), showing that the induction of flowering and senescence can be genetically uncoupled.

While late leaf senescence is mainly likely to improve yield under source but not under sink limitation, there can also be a general benefit of stay-green for water balance, resulting in increased wheat yield under a range of environmental conditions (Christopher *et al.*, 2016). Especially under water-limited conditions, smaller pre-anthesis canopy size in stay-green genotypes improves water relations by reducing transpiration (Borrell *et al.*, 2014; George-Jaeggli *et al.*, 2017). For water-limited conditions, a positive effect of the ratio between post- and pre-anthesis above-ground biomass production (correlated with stay-green and lower canopy size at anthesis) on grain yield was described for sorghum, barley, and wheat (Borrell *et al.*, 2023). This indicates that breeding strategies which reduce pre-anthesis canopy size and water use may be successful by conserving soil moisture during grain filling in environments that are prone to late-season drought. Further, the stay-green trait can be associated with extended root nitrogen uptake, as shown for oilseed rape (He *et al.*, 2021).

While stay-green has mainly been associated with delayed leaf senescence, at the canopy level it can also be achieved through the extended green duration and photosynthetic activity of the inflorescence. Exploring genetic variation in spring wheat, Molero and Reynolds (2020) point out that there were no previous reports of cereal breeding programmes assessing ear photosynthesis systematically. Interestingly, ear photosynthesis was not correlated with flag leaf photosynthesis for individual wheat panels, suggesting that ear photosynthesis can be bred for separately from leaf photosynthesis. Under heat stress, ear photosynthesis was correlated with yield (Molero and Reynolds, 2020), and the photosynthetic apparatus of ears is more resilient to drought stress than that of the leaf (Martinez *et al.*, 2003), supporting a role for inflorescence photosynthesis under stressful conditions. In Mediterranean environments where leaf duration may be limited because of early flowering associated with a drought escape strategy, inflorescence photosynthesis can therefore provide an alternative to traditional leaf stay-green traits (Fig. 2). In common, both stay-green and enhanced inflorescence photosynthesis can extend photosynthetic carbon gain while also conserving water. The difference lies in the timing; stay-green has the potential to conserve water by reducing the canopy size before flowering, whereas increased inflorescence photosynthesis can compensate for reduced leaf area when water becomes limiting later in the season.

Elevated [CO_2_] can to some extent mitigate the effects of climate change by increasing photosynthesis and water-use efficiency. Theoretically, elevated [CO_2_] could therefore contribute to increased source strength. However, a positive fertilization effect of elevated [CO_2_] may not materialize in Mediterranean and other hot and dry climates (Gray *et al.*, 2016; Thomey *et al.*, 2019; Bloom and Plant, 2021). Approaches that increase climate resilience are therefore also required under elevated [CO_2_] conditions. Gross photosynthesis in ears has been shown to respond strongly to elevated [CO_2_], which can contribute to an increase in yield (Gámez *et al.*, 2020), adding to the potential of inflorescence photosynthesis to make an increased contribution to crop carbon gain in a future environment.

## Conclusion

In the changing climate, heat and drought conditions are expected to become more frequent and more extreme. This may require breeding approaches optimized for stressful environments even in regions that in the recent past have had mild climatic conditions, suitable for high yields. For example, temperatures in Europe have been increasing more than twice as fast as the global average (World Meteorological Organization, 2022) and, despite overall high yields, negative impacts of climate change are already a reality, for example for wheat production in France and Germany (Helman and Bonfil, 2022). Future grain production may therefore require approaches that help maintain photosynthesis under stress conditions and overcome source limitation resulting from early flowering to avoid terminal drought. These include enhanced inflorescence photosynthesis, or a version of stay-green that also improves water balance through enhanced root development and reduced water use. In addition, functional stay-green can provide photosynthate as an energy source for extended root nitrogen uptake. Prolonged root nitrogen uptake to support grain filling and protein synthesis is also a suitable breeding target for favourable conditions. Under conditions resulting in terminal drought and low nitrogen availability, fast and efficient senescence-dependent nitrogen recycling is important to support first inflorescence photosynthesis and then grain protein synthesis. Breeding should therefore involve the monitoring of phenological events (flowering and senescence) combined with green biomass accumulation, photosynthetic function of different organs, and grain yield. This can be supported by remote sensing approaches in combination with machine learning for high-throughput phenotyping in the field and yield prediction. For example, thermal imaging can be used to determine plant (Gracia-Romero *et al.*, 2023) and ear (Fernandez-Gallego *et al.*, 2019) water status, while hyperspectral canopy reflectance can be imaged to screen for senescence dynamics and predict grain yield and GPC (Anderegg *et al.*, 2020). Application of these approaches for the phenotyping of field-grown crops allows identification of critical performance traits under realistic environmental conditions for a given climate.

## Abbreviations

GPC: grain protein content
HI: harvest index
NUE: nitrogen-use efficiency
QTL: quantitative trait locus
